# Programmable gradational micropatterning of functional materials using maskless lithography controlling absorption

**DOI:** 10.1038/srep15629

**Published:** 2015-10-22

**Authors:** Yushin Jung, Howon Lee, Tae-Joon Park, Sungsik Kim, Sunghoon Kwon

**Affiliations:** 1 Institutes of Entrepreneurial BioConvergence, Seoul National University, Seoul 151-742, South Korea; 2 Department of Electrical and Computer Engineering, Seoul National University, Seoul 151-744, South Korea; 3 Nano Systems Institute, Seoul National University, Seoul 151–744, South Korea; 4 Interdisciplinary Program for Bioengineering, Seoul National University, Seoul 151-744, South Korea; 5 Seoul National University Hospital Biomedical Research Institute, Seoul National University hospital, Seoul 110-744, South Korea

## Abstract

The demand for patterning functional materials precisely on surfaces of stimuli-responsive devices has increased in many research fields. *In situ* polymerization technology is one of the most convenient ways to place the functional materials on a desired location with micron-scale accuracy. To fabricate stimuli-responsive surfaces, controlling concentration of the functional material is much as important as micropatterning them. However, patterning and controlling concentration of the functional materials simultaneously requires an additional process, such as preparing multiple co-flow microfluidic structures and numbers of solutions with various concentrations. Despite applying these processes, fabricating heterogeneous patterns in large scale (millimeter scale) is still impossible. In this study, we propose an advanced *in situ* polymerization technique to pattern the surface in micron scale in a concentration-controlled manner. Because the concentration of the functional materials is manipulated by self-assembly on the surface, a complex pattern could be easily fabricated without any additional procedure. The complex pattern is pre-designed with absorption amount of the functional material, which is pre-determined by the duration of UV exposure. We show that the resolution reaches up to 2.5 μm and demonstrate mm-scale objects, maintaining the same resolution. We also fabricated Multi-bit barcoded micro particles verify the flexibility of our system.

*In situ* polymerization is one of the most convenient ways to fabricate microstructures in microfluidic environment by photopatterning liquid-phase photocurable materials^1^,^2^,^3^,^4^,^5^. Recently, use of dynamic mask in the maskless lithography technique dramatically improved flexibility and performance of the *in situ* polymerization, opening broad research areas^3^,^6^,^7^,^8^. This technique allows fabricating not only the microstructures by photo-curing a single type of photopolymer, but also functionalized microstructures by incorporating functional materials, such as cells or photochromic materials, in the photo-curable polymer^6^,^8^,^9^,^10^.

In certain applications, concentration gradients or steps of functional materials are essential for optimizing the overall efficiency such as optimizing physical/chemical properties of polymers, discovering drugs, and growing neural stem cells while maintaining low production costs and minimizing research effort^11^,^12^,^13^,^14^. In the past, researchers produced concentration gradients by adjusting target material deposition or by dipping the substrate in target materials after templating^15^,^16^,^17^,^18^,^19^. These methods are based on manipulating unidirectional gradation patterns, without the ability to fabricate complex microstructures with discontinuous concentration steps. In a general *in-situ* polymerization approach, multiphase flow of polymer solutions (each mixed with desired concentrations of functional materials) is used to design concentration steps. The laminar flow, formed by this multiphase flow, develops sharp interfaces between different solutions in a microfluidic environment. Using this phenomenon, microstructures with multi-step concentrations of functional materials can be formed^3^,^5^,^20^,^21^.

However, there are four major limitations of these approaches for practical purpose. First, because multiphase should be generated at the microfluidic circumstance, conventional *in situ* polymerization technique is not appropriate for large-scale production. Second, microfluidic structures become more complicated as they require diverse concentrations. Moreover, due to the continuous flow, huge portion of waste is generated which includes expensive ingredients. Most of all, it is impossible to fabricate complicated patterns with the controlled concentration using laminar flow.

In this study, we propose an advanced *in-situ* photopolymerization technique, applying post-deposition to construct aheterogeneous micro-patterned functionalized polymer with multi-level concentration. Our technique, named Maskless Lithography Controlling Absorption (MLCA), is based on well-established maskless grayscale lithography^22^,^23^. We first polymerizes host polymer with multilevel UV exposure. Because the maskless lithography provides dynamic micropatterns that are generated relatively fast through the custom-designed software, the multilevel UV exposure can be determined with accumulation of sequential exposure patterns. The multilevel UV exposure can controllably produce a variety of cross-linking densities^24^,^25^,^26^. Therefore, the polymer-based functional material penetrates with diverse pre-determined concentrations to the host polymer, which is then templated with a variety of cross-linking densities. Since the MLCA technique controls concentration difference by multilevel UV exposure only, preparing reagents in different concentrations is not required. Therefore, large-scale and highly heterogeneous micropattern fabrication is possible without any complex experimental setup and procedure. In addition, waste can be reduced for cost effective manufacturing.

We micropatterned the UV-triggered photochromic functional material (1′,3′-dihydro-1′,3′,3′-trimethyl-6-nitrospiro[2H-1-benzopyran-2,2′-(2H)-indole], or spiropyran) with 2.5 μm resolution in multiple concentrations to demonstrate our technique. We chose spiropyran because of its interesting property that it reversibly transforms from transparent to purple when exposed to UV light^10^,^27^,^28^,^29^,^30^,^31^,^32^. Moreover, reversible change of the physical properties of photochromic materials attracts researchers for designing practical applications. For new applications, micropatterning the photochromic material to an appropriate location with various concentrations is essential^10^,^33^. Spiropyran also has a fluorescent property that enables analyzing absorption optically^27^,^30^,^32^. We demonstrated free-floating micro-particles and large-scale (up to millimeters) micro-patterns using a step and repeat method to show the technical advantage of this method over conventional micro-patterning approaches.

## Result and Discussion

**Figure 1:**
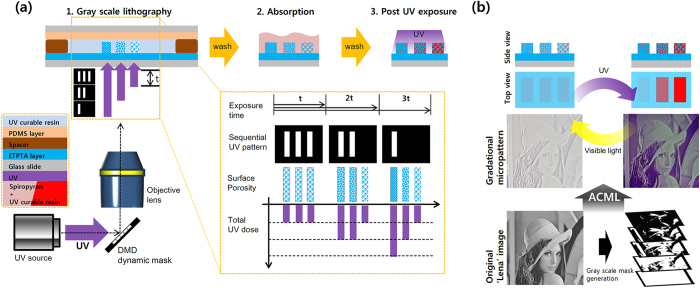
Fabrication of gradational micropattern. (**a**) Overall workflow of MLCA is composed of three steps: 1) Grayscale lithography, 2) absorption, 3) Post-UV exposure. Crosslinking density varies according to exposure time. (**b**) Demonstration of gradational micropattern using MLCA system. Spiropyran maintained photochromic characteristics (changing color depending on exposed light wavelength) while absorption is controlled.

To demonstrate the gradational deposition of spiropyran, we chose the familiar ‘Lena’ image, which has been widely used as an example in image processing for a long time (Fig. 1). The photocurable resin (ethoxylated trimethylolpropane triacrylate (ETPTA) mixed with photoinitiator) was loaded between two different glass slides; one was coated with thin layer of ETPTA to hold the microstructure and the other was coated with a polydimethylsiloxane (PDMS) layer to prevent adhesion of microstructure (Fig. 1a). The spacer determines the maximum thickness of the microstructure. UV light was projected by reflecting from a spatial light modulator, digital micromirror device (DMD), and focused onto the surface of the ETPTA-coated glass slide to cure the host polymer. The DMD was used to vary the UV dosage at each location at a micron scale for multi-step exposure. The DMD generates dynamic sequential masks; five masks were made by processing 256-scale gray image by adjusting appropriate threshold values (see Supplementary Fig. S1 online). By sequentially patterning these five masks with same time interval, total UV exposure was varied spatially depending on the number of the binary mask that patterns a certain spot, and thus the UV dose could be controlled (the inset of Fig. 1a). After washing out the uncured polymer, spiropyran was absorbed to the cured polymer. Unabsorbed spiropyran was washed out. Absorption of spiropyran depends on the cross-linking density of the host polymer, which is controlled by the total dose of UV; position with higher UV dosage appeared to absorb less spiropyran and vice versa (Fig. 1b). Detailed fabrication processes and materials are included in Methods section and Supplementary Fig. S2.

**Figure 2:**
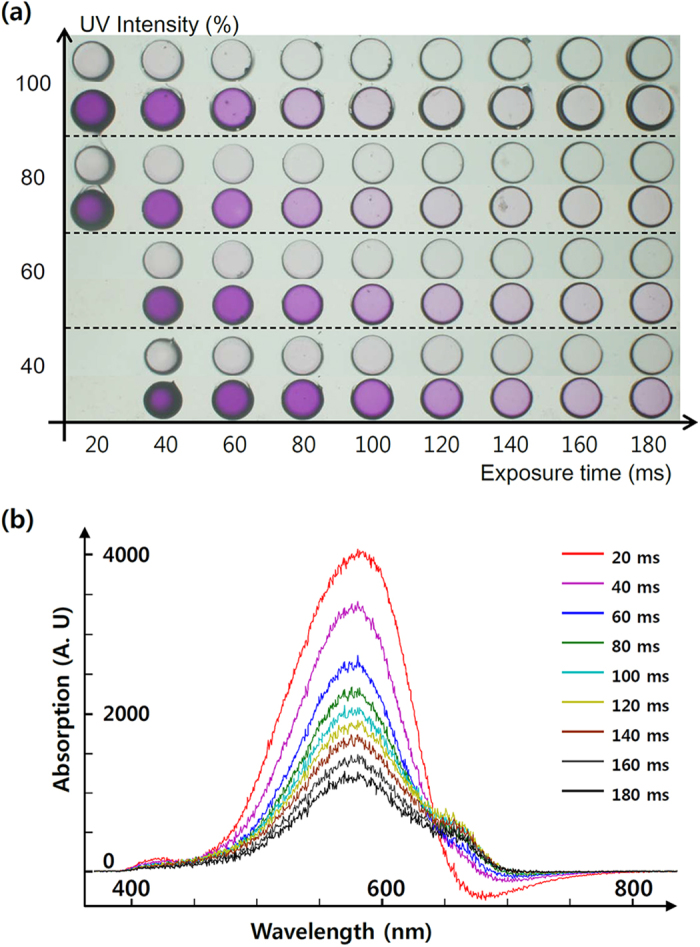
Optical analysis of ETPTA-hosted spiropyran absorption. (**a**) Gradational color map of a circular spiropyran micropattern with varying UV-exposure intensity (40 ~ 100%, where intensity 100% corresponds to 180 mW/cm^2^) and time (20 ~ 180 ms). Reduced UV dosage results in a stronger purple color. (**b**) Absorption spectra of micropatterns with a fixed UV intensity (80%), with variation in exposure time. Negative value in the longer wavelength region (red) signifies a fluorescence signal of the spiropyran molecule.

Absorption of spiropyran depends on the UV exposure intensity and duration while curing the host polymer (Fig. 2). We used varying UV exposure intensity from 40 to 100% (180 mW/cm^2^) and exposure time ranged between 20 and 180 ms (Fig. 2a). As the exposure time increased for a given intensity, the purple color of absorbed spiropyran gradually diminished. Similarly, higher intensity caused the color to diminish at a given exposure time. Excessive UV dose ruined the details in the micropattern due to radical diffusion and light overlap. Underexposed micropatterns were lost during the washing step. Consequently, an appropriate UV-exposure intensity was determined to range from 80 to 100%, with an exposure time ranging from 25 to 150 ms. Absorption varied depending on the exposure time, in a broader scale than that of the intensity. Therefore, we decided to control total UV dose solely through variation in exposure time. We used absorbance spectroscopy for more delicate measurement of the absorbed spiropyran concentration (Fig. 2b). Absorbance spectrum data show negative correlation between exposure time and the absorption intensity. This implies that controlling concentration gradation level on a continuous basis is enabled by very high switching speed (~100 μs) of the commercial DMD.

**Figure 3:**
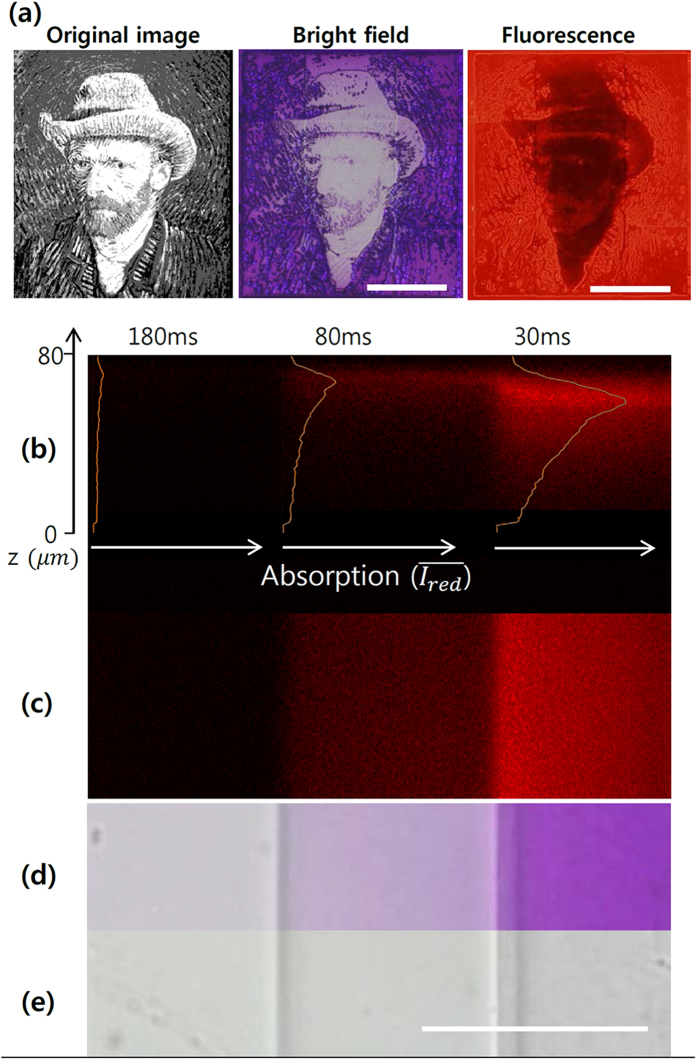
Fluorescence microscope image. (**a**) Bright field and fluorescence image of “*Self-Portrait with Grey Felt Hat”,* Vincent van Gogh, 1887/88 (Scale bar: 500 μm). (**b–e**) Confocal fluorescence observation of absorption characteristics of spiropyran in an ETPTA polymer matrix (Scale bar: 100 μm); (**b**) side view, (**c**) top view, and (**d**) top view in bright field microscopy image after exposure to UV light and (**e**) to normal light.

Confocal microscopy was used to investigate the three-dimensional absorption characteristics of microstructure fabricated by our MLCA method. Fluorescence emission of the colored merocyanine form of spiropyran showed red color from fabricated patterns, which are purple in bright field (Fig. 3a). Three-dimensional confocal imaging was conducted on the assumption that all absorbed spiropyran molecules fluoresce equally inside the ETPTA polymer. Three different concentrations of spiropyran were achieved by 180, 80 and 30 ms of 100% intensity of UV exposure (Fig. 3b–e). As expected, the shorter exposure time resulted in a stronger purple color in bright field microscopy (Fig. 3d). The top-view confocal microscopy image showed a step-wise increase in fluorescence, which is consistent with the bright field image (Fig. 3c). Side view of the structure shows that spiropyran absorption quantity permeated from the surface to a depth of 80 μm polymer mesh (Fig. 3b). The fluorescence intensity and concentration of absorbed spiropyran are highest at the surface and gradually decrease with depth. This is mainly because of oxygen from the surrounding air freely diffusing through the porous PDMS layer. The oxygen permeability of PDMS is well-known, and we have applied this phenomenon to free-floating polymeric particle fabrication with PDMS microfluidic device^3^,^5^,^6^,^7^,^8^,^20^,^21^. However, the application of UV light causes cross-linking to begin at the ETPTA-coated glass slide surface, from which the oxygen-supplying PDMS wall is farthest away. The presence of oxygen also hinders the free-radical photopolymerization reaction by chain termination^24^. Because oxygen is not able to diffuse through the glass slide, polymerized structure growth occurs toward the PDMS wall while consuming oxygen. When one area has been sufficiently radiated by UV, the microstructure is filled with a fully cross-linked solid network and stops growing, and a thin space is generated between the structure surface and the PDMS wall. We anticipate that UV dose could control cross-linking density, which decides the quantity of permeating spiropyran. For example, if the cross-linking density of the generated structure is high, spiropyran would barely permeate to the resultant structure with low porosity. Also in case of an insufficient UV dose, photopolymerization would stop and leave a less cross-linked surface on the PDMS wall side. Thus, the structure would absorb much more quantity of spiropyran compared to the fully cured microstructure. Subsequently, most of spiropyran absorbed in a UV-dosed microstructure fabricated by the MLCA method tended to stay near the surface.

**Figure 4:**
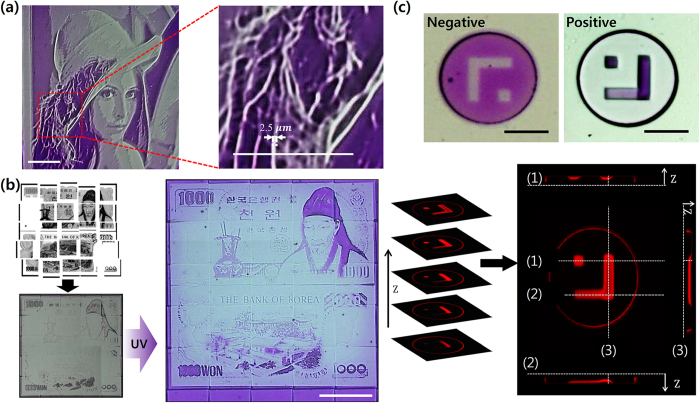
Various examples of gradational micropatterning products. (**a**) Maximum resolution verified by the feather micropattern of Lena’s hat, with a 100 μm scale bar. (**b**) Large-scale spiropyran micropattern fabrication based on a step and repeat method (Scale bar: 500 μm). ((**c**) top) Microparticles with orthogonal and gradational spiropyran micropattern and ((**c**) bottom) a confocal image of gradational spiropyran deposition at a predetermined location and concentration (Scale bar: 100 μm).

The maximum resolution of the MLCA system was validated through one of our examples: the ‘Lena’ micropattern. When we compared the thin-line pattern of the feather with the scale bar located on the bottom of the image (200 μm), the expression ability of the MLCA system reached at least 2.5 μm (Fig. 4a). This result is certainly comparable to the findings of previous studies and suggests an immediate applicability^34^,^35^,^36^,^37^. Furthermore, because the MLCA method projects patterns through an objective lens (10×, NA 0.30), a more precise micropattern is achievable by simply using a lens with a higher numerical aperture.

Because of the flexibility of the MLCA system, various types of photochromic microstructures have been demonstrated for a variety of applications. Applying a ‘step and repeat’ approach, large-scale spiropyran micropatterning was performed with the help of a motorized stage and custom software. Korean 1000 won bill image was divided into 5×5 pieces for the step and repeat MLCA system fabricating 2-mm sized structure (Fig. 4b). Because each single exposure maintains high resolution, fine details such as roofing tiles and leaves of trees can still be observed. Although the fabrication of a large-scale absorption-controlled pattern would require multi-location exposure, the MLCA method only requires a single absorption step to deposit the spiropyran. Consequently, the complexity is less when compared to a traditional step and repeat method. Using gradational deposition and DMD projection lithography, we also demonstrated a photochromic multi-bit barcoded microparticle with an arbitrary shape (Fig. 4c). Multi-bit barcoding offers a much higher capacity over conventional binary barcoding^6^; suppose there are “n” number of barcoding spots and we decide to use “m”-ary bit, the barcoding capacity increases to *m*^*n*^ by using our technology, compared to 2^*n*^ when barcoding with binary codes. Spiropyran can also act as a probe site for the capture and release of DNA or metal ions absorbed in microparticles^27^,^29^.

In conclusion, we have proposed an advanced photochromic material deposition method suitable for precise gradational micropatterning, based on the MLCA technique. The proposed method combines high precision of lithography with selective gradational self-assembly of polymer-hosted spiropyran in a cost effective and scalable manner. Therefore, this technique enabled us to fabricate a complex micropattern with four different concentrations of spiropyran with 2.5 μm spatial resolution. In addition, it enabled the fabrication of large-scale (millimeter) structure by applying the step and repeat method maintaining high resolution. Finally, a multi-bit barcoded microparticle was fabricated to demonstrate the flexibility of our system. Further optimization would provide much higher resolution. Through the creation of these various photochromic microstructures, a physiochemical analysis of the MLCA technique was conducted, validating our approach. We show that a well-established maskless lithography for grayscale-patterning technique could be applied to photochromic materials. We believe that the MLCA technique would broaden the application of photochromic materials to fields such as displays, molecular sensors, bioassay, and anti-counterfeiting. Furthermore, because the MLCA technique can use various functional polymers that are dissolvable in photocurable polymer, this technique has the potential to be used as a universal tool for functional materials.

## Methods

### Absorption-controlled micropattern fabrication

The polydimethylsiloxane (PDMS)-coated glass was made by spin-coating PDMS with 10 wt% of curing agent. For the photocurable polymer solution (A) Ethoxylated Trimethylolpropane Triacrylate (ETPTA, MN = 428) with 5 wt% of photoinitiator (2, 2-dimethoxy-2phenylacetophenone, DMPA) was used (B). A micropattern with multi-degree porosity was generated by exposing micropatterned UV light sequentially with different intensities and exposure periods onto (A) which is in between the PDMS-coated glass and the glass in certain spacer through objective lens (10×, NA 0.30, infinity–/– FN 26.5, Olympus). Micropatterns were generated by converting the original image to several binary images using given thresholds, and these patterns were loaded sequentially on a digital micromirror device (DMD, Texas instruments) to generate micro-patterned UV light by using our custom-designed software. The 100% intensity of UV light corresponded to 180 mW/cm^2^, and this intensity is thought to be decreased proportionally as the percentage decreases.

### Polymer host photochromic materials absorption

Photochromic material (1′,3′-Dihydro-1′,3′,3′-trimethyl-6-nitrospiro[2H-1-benzopyran-2,2′-(2H)-indole]), which was referred as spiropyran in this paper, was mixed with host polymer (A) at a concentration of 4 wt%. Absorption was found to take several minutes, with residual photochromic material removed by a strong N_2_-gas blow and a gentle EtOH wash.

### Observation method

Bright field images were obtained using an optical microscope (IX 71, Olympus) equipped with a true-color charge-coupled device (CCD) camera (DP72, Olympus). Fluorescence images were obtained using an optical microscope with fluorescence filter cube (U-MWG2, Olympus). Confocal images were obtained by excitation with a 543 nm laser in the red spectral detection range (DMI6000, Leica).

## Additional Information

**How to cite this article**: Jung, Y. *et al.* Programmable gradational micropatterning of functional materials using maskless lithography controlling absorption. *Sci. Rep.* **5**, 15629; doi: 10.1038/srep15629 (2015).

## Supplementary Material

Supplementary Information
