# Supplementary material for: Programmable gradational micropatterning of functional materials using maskless lithography controlling absorption
Source: Sci Rep. 2015 Oct 22;5:15629. doi: 10.1038/srep15629 (PMC4615026; doi:10.1038/srep15629)
Supplement: Supplementary Information [file srep15629-s1.pdf]

Supplementary information

Programmable gradational micropatterning of functional materials using maskless lithography controlling absorption

Yushin Jung[1, 2]§, Howon Lee[1]§, Tae-Joon Park[1], Sungsik Kim[1, 3], and Sunghoon Kwon[1, 2, 4*]

§ Both authors equally contributed to this work.

[1]Institutes of Entrepreneurial BioConvergence, Seoul National University, Seoul 151-742, South Korea

[2]Department of Electrical and Computer Engineering, Seoul National University, Seoul 151-744, South Korea

[3]Interdisciplinary Program for Bioengineering, Seoul National University, Seoul 151-744, South Korea

[4]Seoul National University Hospital Biomedical Research Institute, Seoul National University hospital, Seoul 110-744, South Korea

*Correspondence to: skwon@snu.ac.kr

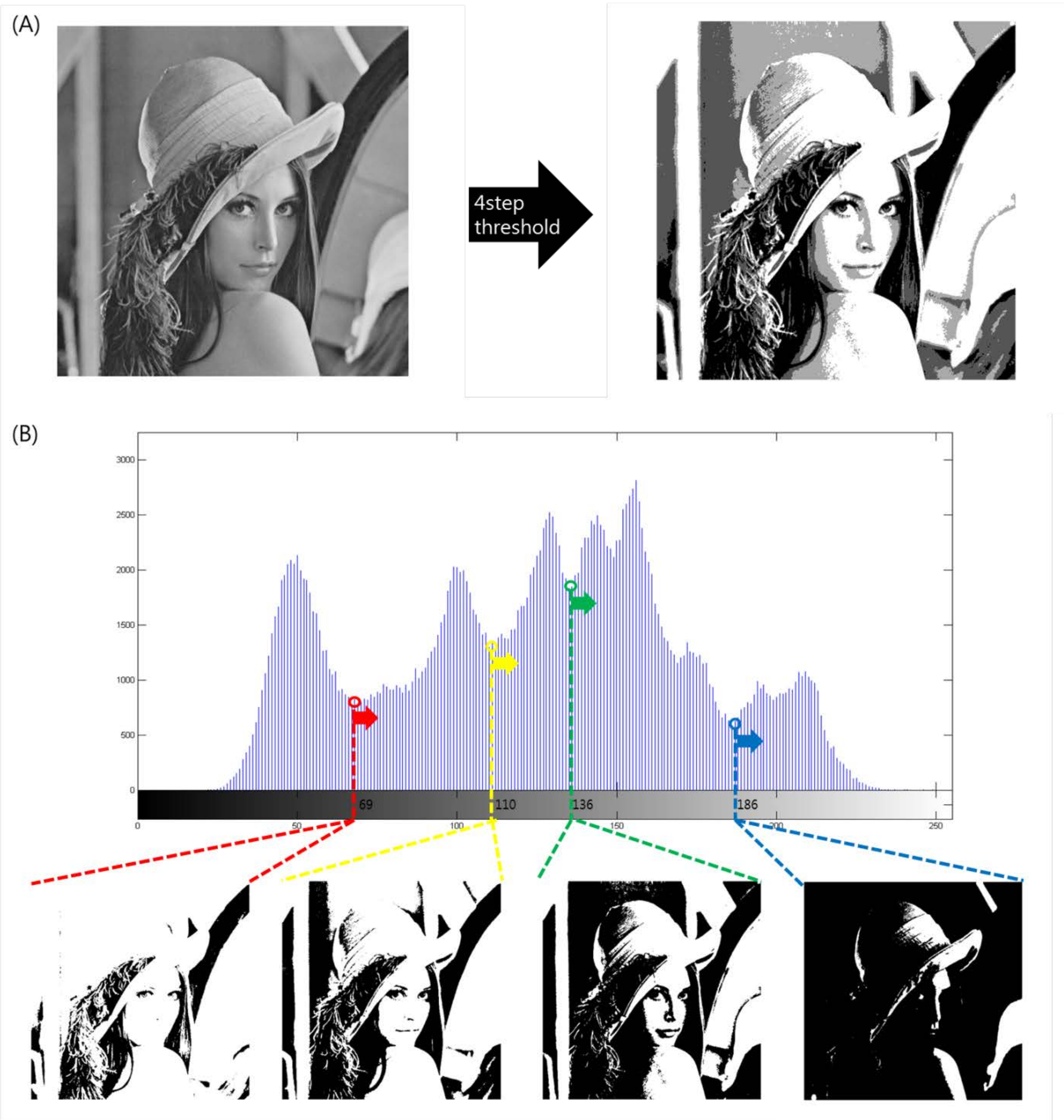

Figure S1. Gray scale mask generation from original image with 4 threshold values (A) Original Lena image (left) and sum of four Lena's binary images divided by four thresholds (right). (B) Four binary images, using function called 'im2bw' in 'MATLAB' which changes gray image to black and white image according to threshold; for example if certain pixel's intensity is less than threshold it makes it black and makes it white in opposite case. We loaded totally five masks including base mask (whole white) to generate figure.

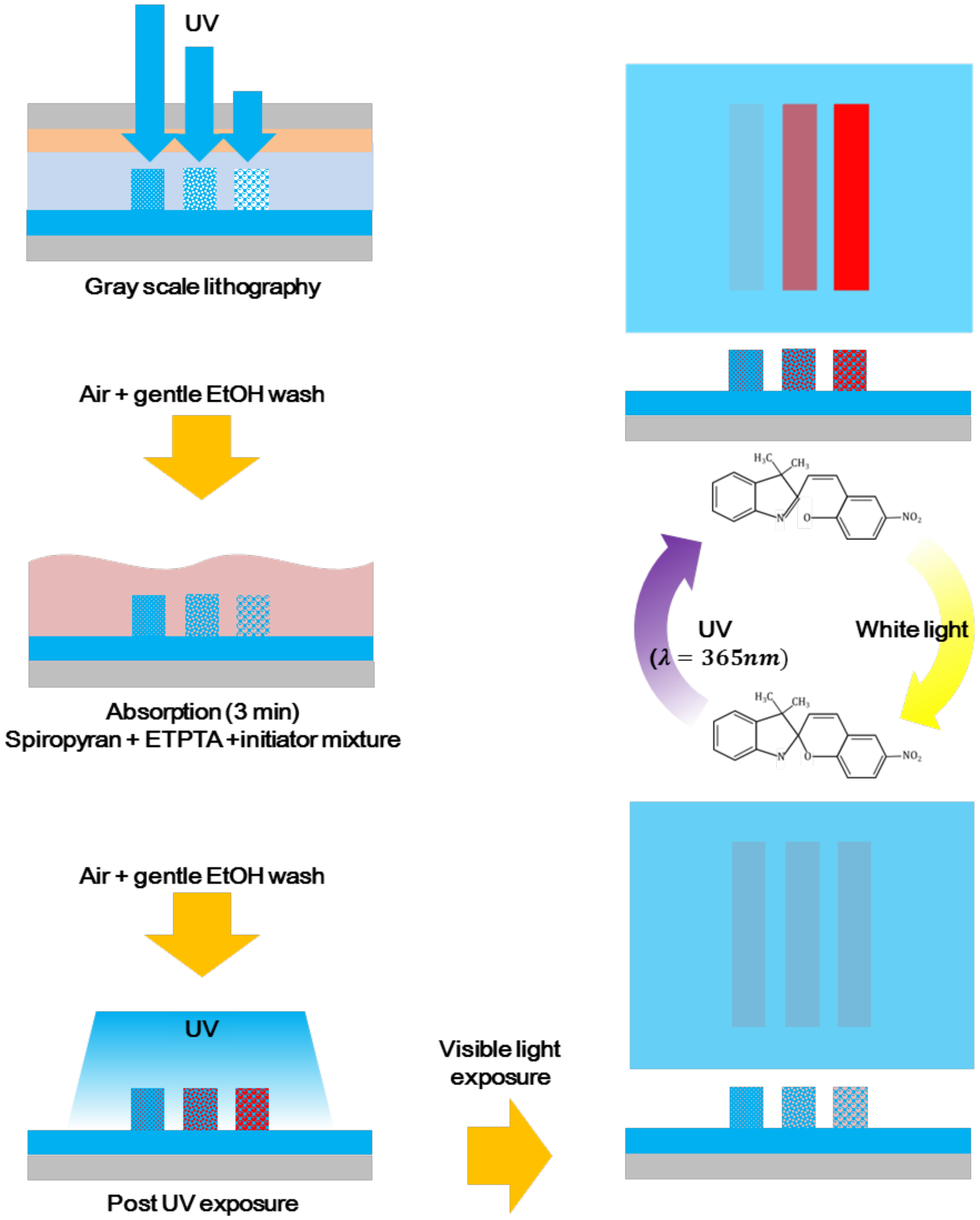

Figure S2. Flow chart for the fabrication of a gradational spiropyran micropattern, using grayscale lithography to produce a variable degree of porosity. Strong blowing with N_2 gas, followed by a gentle EtOH wash, was used to remove uncured residual monomers and initiator both inside and outside the microstructure. Spiropyran is then absorbed for several minutes, with the least dosed parts of the micropattern having the greatest absorption owing to their greater porosity. Excess spiropyran was then removed by further washing with N2 gas and EtOH. Subsequent UV exposure is required to stabilize both the polymer mesh and the absorbed spiropyran; however, an overlap exists in the absorption spectra required for each necessitating additional time beyond that required for manufacturing. As a result of the variation in absorption rate, different parts of the resultant microstructure show a varying intensity of purple coloration after UV exposure that can be reversibly turned on and off by sequential radiation of UV and visible light.

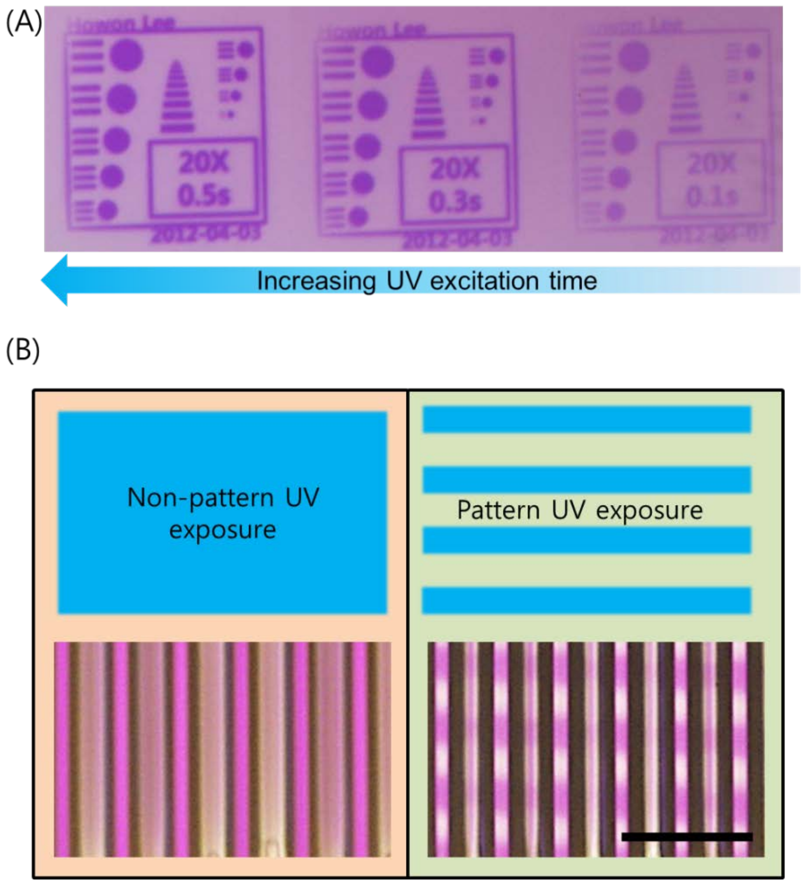

Figure S3 Excitation characteristics of ETPTA host spiropyran with different excitation time and pattern. (A) Intensity of spiropyran color increase as UV excitation time increases. UV pattern generated from DMD. (B) Non-pattern UV exposure let spiropyran micropattern display purple color all over them while stripe pattern UV exposure selectively excite spiropyran that only target region shows purple color. Pattern UV with various exposure times shows huge potentials of our product to gradational display and anti-counterfeiting. (Scale bar: 100μm)
